# Nickel Foam Electrodes—A Versatile, Powerful, and Readily Available Tool in Electro‐Organic Synthesis

**DOI:** 10.1002/tcr.70152

**Published:** 2026-04-18

**Authors:** Rok Narobe, Johannes Schneider, Siegfried R. Waldvogel

**Affiliations:** ^1^ Department of Electrosynthesis Max‐Planck‐Institute for Chemical Energy Conversion Mülheim an der Ruhr Germany; ^2^ Faculty of Chemistry and Pharmacy Institute of Organic Chemistry University of Regensburg Regensburg Germany; ^3^ Karlsruhe Institute of Technology (KIT) Institute of Biological and Chemical Systems – Functional Molecular Systems (IBCS–FMS) Karlsruhe Germany

**Keywords:** anode, cathode, electrolysis, metal foam, reduction

## Abstract

For more than 50 years, nickel foam electrodes have served in electrosynthesis as powerful and readily available electrode materials. Due to their inexpensive nature and easy handling, they have been widely employed. Their application as an active anode material, which generates a conductive and insoluble layer of NiOOH in alkaline media, gives access to a selective oxidizing system. While using NiOOH anodes as an electrocatalyst has already emerged in the past and was rediscovered lately, the use of nickel foam electrodes as a highly active cathode material for the reduction of organic compounds has strongly evolved only within the last decade. Electroreductions on nickel foam can be carried out in both alkaline and acidic media, as well as under protic and aprotic conditions, enabling a broad range of transformations. The affordability, high electrical conductivity, large electrode surface area, and specific electrocatalytic features are in combination unique and unmatched by any other electrode material. The developments and, in particular, recent breakthroughs for this electrode material in electro‐organic synthesis are surveyed.

## Graphical abstract

Nickel foam—one material, two faces, and several versatile roles depending on electrolytic conditions.

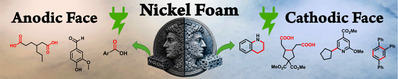

## 1 Introduction

Electrosynthesis is the utilization of electricity to carry out valuable and interesting synthetic transformations via anodic oxidation and cathodic reduction reactions [1, 2, 3, 4, 5, 6, 7, 8, 9, 10]. Electrosynthesis offers a wide range of advantages over conventional chemical transformations, which is the reason why this field is currently experiencing a tremendous renaissance to establish itself as a 21^st^‐century technique [11, 12, 13]. The use of stoichiometric amounts of oxidizing and reducing agents is avoided, which also prevents reagent waste. Electrons are used as an inexpensive, universal, and traceless redox reagent. Electrosynthetic transformations are characterized by comparably mild reaction conditions while offering an enormous thermodynamic driving force. Also, electrosynthesis offers unique reactivity and specific selectivity, and there are electrochemical reactions that do not have an analogy in conventional synthesis [14, 15, 16]. This is especially interesting when it's employed as a technique for late‐stage C–H functionalization [17]. In addition, electrosynthetic transformations are usually readily scalable [18]. Electrochemical transformations are characterized by inherent safety, because electricity can be switched off, and then the conversions cease immediately. Another advantage is the adaptive power consumption, which enables the use of intermittent electricity [19, 20, 21, 22]. What electrode material to choose as anode and cathode is a central parameter of utmost importance for electro‐organic synthesis [23, 24, 25], because electrode materials differ in their physical properties, which directly affect the envisioned electrochemical transformation [26]. When employing metals as cathode materials, cathodic corrosion must be prevented [27]. When employed as an anode material, nickel should normally dissolve under oxidative conditions. However, there are several nickel salts that are readily generated via electrolysis that form a compact and insoluble layer on the anode surface, which exhibits good electrical conductivity. Noteworthy are, among others, nickel oxide hydroxides and nickel fluorides. They have already been recognized in the past and even led commercial operations such as the vitamin C synthesis by oxidation of L‐sorbose [28], or the Simons fluorination process to polyfluorinated compounds [29]. Nickel foam electrodes are employed as a cathode material in the sewage treatment of wastewater originating from industrial electroplating for the recovery of noble metals [30] or for an electro‐Fenton treatment (generation of hydroxyl radicals) of organic pollutants [31, 32].

### 1.1 Outline of This Review

With this review, we aim to present specific practical features and versatile roles of nickel foam electrodes in electro‐organic transformations. The review starts with a chapter about practical tips and continues with examples of synthetically valuable transformations, which are divided into two main parts—anodic and cathodic transformations. In the anodic part, we present alcohol and aldehyde oxidations, olefin epoxidations, and oxidative C—C bond cleavages achieved by NiOOH functionalized Ni foam. In the cathodic part, we present examples of hydrogenation of olefins, nitriles, N‐heterocycles, carboxylation reactions of olefins, organic halides, and different cross‐coupling reactions mediated by nickel or cobalt catalysts.

Nickel foam is also a widely used support template for engineered catalytic deposits (e.g., nanosheets and nanowires), and such materials have indeed found applications in organic synthesis. However, these advanced catalytic systems are only loosely related to the nickel foam support itself. In this review, we therefore focus on simple, commercially available, or easily prepared materials. Readers interested in catalytic deposits on nickel foam are directed to the following reviews and selected studies [32, 33, 34, 35, 36, 37, 38, 39, 40, 41].

## 2 Practical Aspects

### 2.1 Specific Features of Nickel Foams

Nickel is an earth‐abundant, readily available, and inexpensive electrode material. The main feature of nickel foam electrodes is the very large electrochemically active surface area in relation to the geometric dimensions of the electrodes, which directly influences the heterogeneous electron transfer at the electrode surface [42]. Because of its emerging use and versatile applications, several producers of nickel foam electrodes were established. These provide pure nickel foam or chemically deposited nickel, which can be considered to be a nickel‐phosphorous alloy. Nickel foams are mechanically stable, nonpyrophoric, and commercially available in various dimensions. These features are advantageous compared with other porous but often brittle electrode materials such as reticulated vitreous carbon (RVC) [23].

### 2.2 Nickel Foam Manufacturing

Several methods exist for nickel foam production to achieve the desired porosity, electrode size, and density [43, 44, 45]. Nickel foam is most commonly produced by using a well‐established polymer foam replication method. In this process, a porous template made from a polymer material is chosen to define the desired foam structure. Nickel can be deposited chemically on the foam template by using various phosphorous‐based reducing agents such as hypophosphite, leading to Ni‐P deposits. These deposits are electrically conducting, allowing for the electroplating of nickel on the surface. The remaining polymer material is removed by chemical dissolution, followed by sintering of the electrode. The most common porosity is 0.4 mm, though in many works, it is not reported which pore size and thickness were used. To achieve the desired electrode shape, the foam can be cut with a carpenter's knife or different types of electric saws. It is important to avoid cutting it with less sharp objects like scissors, as they bend and squeeze the foam and thus influence the local current density distribution on the surface of the foam. In addition, this can negatively affect mass transport, both overall and locally. Some groups perform a short pretreatment of the foam electrodes directly prior to electrolysis to remove oxides from the nickel surface [46]. We recommend avoiding sonication of nickel foam electrodes because this can damage them. In most cases, direct use without pretreatment is viable.

### 2.3 Electrolysis using Nickel Foam Electrodes

**FIGURE 1:**
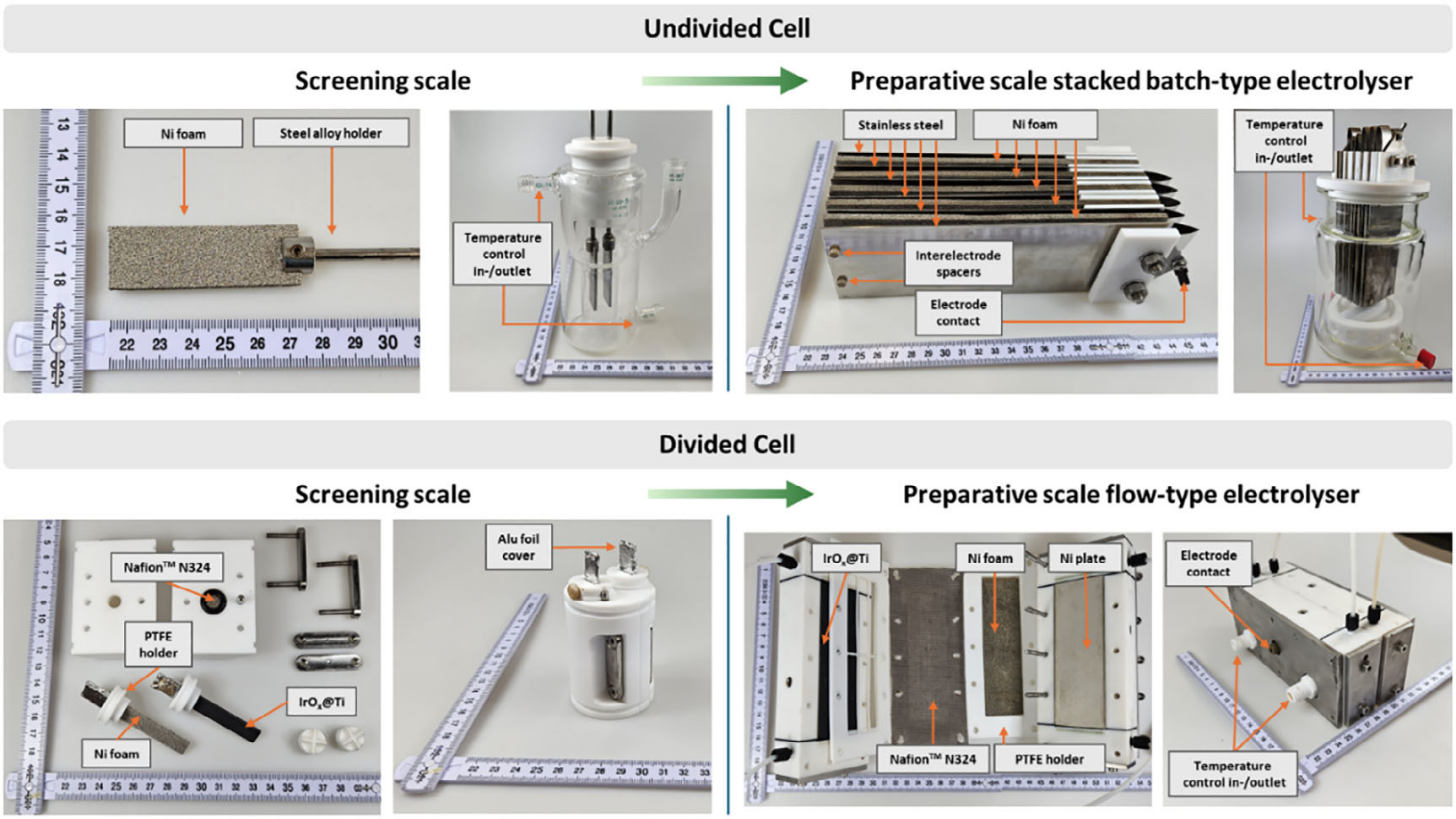
Examples of experimental setups from Waldvogel Lab. Undivided jacketed glass cell setups are marketed as SynLectro and are commercially available from Merck S.A. Divided batch and flow electrolyzers are commercially available from IKA. Ruler scales are in centimeters.

When employing foam electrode materials, the electric current density can only be approximated as geometric current density, which is the approximate surface area of the electrode in solution that is facing the counter electrode. This is because the electrochemically active surface area during electrolysis is not known and not trivial to measure. It's also subject to change during electrolysis, especially in the case of NiOOH electrodes. In general, an electrosynthesis publication should give as many details as possible on the electrodes. In the case of nickel foam electrodes, the pore size must be reported. Although nickel foam electrodes are suitable for a wide range of electrolyzer configurations, several important considerations must be taken into account when employing them (Figure 1). In electrolysis cell designs in which the electrode mounting is in contact with the reaction mixture (fully immersed electrode), the holder should be made out of nickel or highly alloyed stainless steel to avoid corrosion [47]. Likewise, in a flow setup, the nickel foam should be contacted with a nickel plate electrode. This is a necessary precaution to prevent errors originating from reactions happening on the holder surface or nickel surface modifications due to electro‐deposition of other metals. In batch‐type cells, where a part of the foam sticks out of the electrolyte solution, it is a good practice to cover the exposed part of the electrode with aluminum foil to prevent fast evaporation of the reaction solvent due to capillary suction. Along these lines, it is also important to keep in mind that electrolysis is also happening in those foam pores that are filled with electrolyte above the reaction solution level. After electrolysis, the foam electrodes will be soaked with the reaction mixture. Therefore, rigorous rinsing of the electrode with solvent is required to wash out the reaction mixture from the foam's porous structure. Alternatively, when working with an internal standard, the foam electrode can also be transferred into a separatory funnel along with the reaction mixture. In a flow setup, it is good practice to add the internal standard and to let the reaction mixture cycle through the cell to ensure thorough mixing of the internal standard and the product trapped within the foam structure. This will result in a homogenized reaction solution, from which a representative sample can be taken for analysis. Unlike with planar electrodes, there is no easy and reliable way to clean the foam structure from surface deposits. Therefore, we recommend using a new piece of foam for every reaction during the reaction development. Once the reaction conditions are established and they have been successfully tested for reproducibility, a single piece of foam can be safely reused continuously, for example, in a larger flow electrolyzer. Still, it is good practice to repeat the same reaction from time to time as a control reaction, to be aware of the gradual changes in the activity of the installed foam electrode. In the context of practical consideration of using foam electrodes, we would like to encourage interested readers to read a great viewpoint article by Lee or Huand and Chu about best practices of benchmarking the performance of foam materials [48, 49].

## 3 Nickel Foam as an Anode Material

**SCHEME 1:**
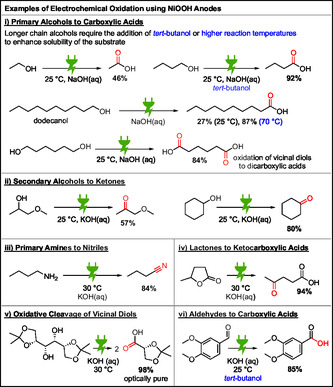
Examples of electrochemical oxidation reactions using NiOOH anodes [28].

Nickel foam electrodes have been extensively applied for various anodic transformations, and a collection of examples is depicted in Scheme 1 [28]. When nickel foam electrodes are employed as anodes in alkaline aqueous media, a layer of nickel oxide hydroxide (NiOOH) is formed and continuously regenerated at the surface of the electrode during electrolysis. This NiOOH on the electrode surface acts as an electrocatalyst for various indirect anodic transformations. Electrodes like these are termed active electrodes since the electrocatalyst is immobilized on the surface. This electrochemical regeneration of the surface avoids the need for stoichiometric amounts of oxidants, as is the case in conventional oxidation reactions. It is advantageous because this layer of NiOOH prevents the dissolution of the electrode material during electrolysis, as is the case for sacrificial electrodes such as aluminum, magnesium, or zinc. Such sacrificial electrodes are not green, because the energy that is required to recover these metals from the reaction waste outweighs their benefit in employing them in electrolysis.

For better reactivity, the protocol of activation is essential for the long‐term performance during operation. The electrocatalytic mode of oxidation is one of the major advantages of NiOOH electrodes. The reactivity of an NiOOH anode is best compared to nickel peroxide. However, the preparation of nickel peroxide is comparably complicated. Therefore, NiOOH anodes have proven themselves as an inexpensive and versatile electrochemical alternative.

### 3.1 Activation of Nickel Foam Anodes

**FIGURE 2:**
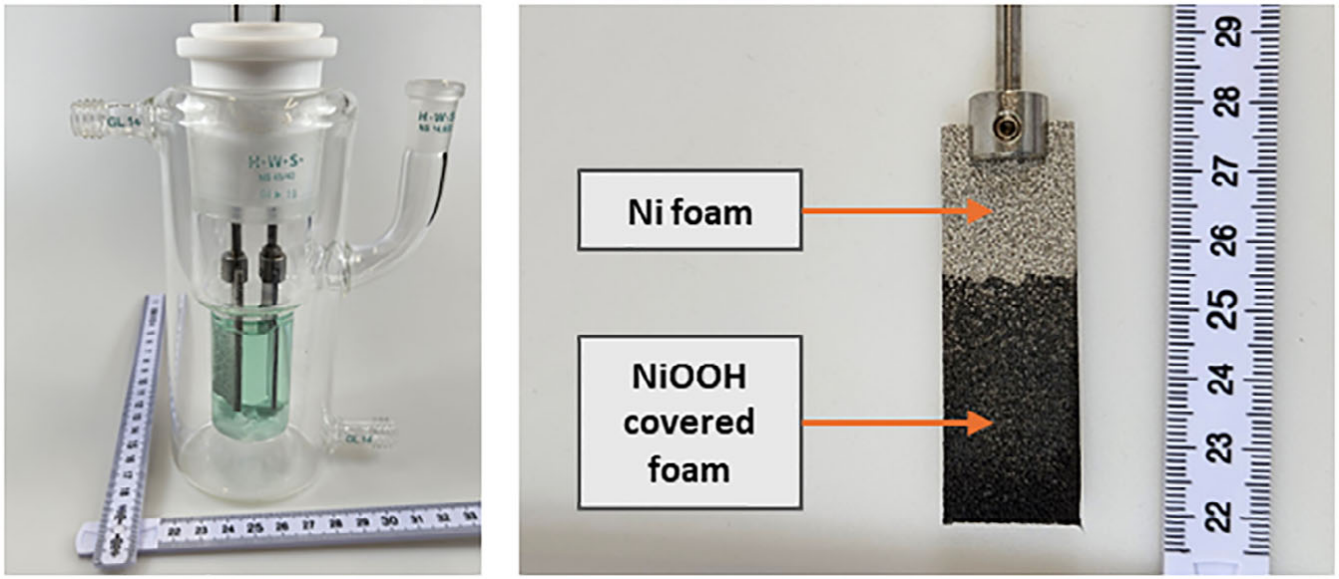
Activation of nickel foam electrodes in aq. NiSO_4_ and NaOH. The NiOOH layer is easily distinguished from unfunctionalized foam by its dark appearance.

**SCHEME 2:**
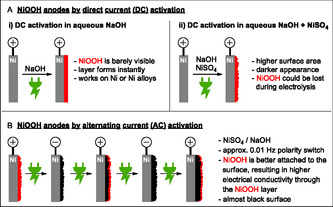
Activation methods for the preparation of NiOOH anodes.

The pretreatment of the nickel foam anode is very important for its activation, which affects the resulting electrocatalytic performance. A common method is to pretreat the nickel electrodes in an aqueous alkaline nickel sulfate buffer solution (activation solution) using a constant current electrolysis under change of polarity (approximately every few minutes) of the electrodes, which leads to the deposit of a thin layer of NiOOH on the nickel electrode, which is more porous and better contacted to the nickel electrodes since part of the NiOOH has been reduced to the metal [50, 51]. Usually, the attachment of NiOOH by this method is better than in other pretreatment protocols (Scheme 2 and Figure 2).

The pretreated electrodes are then employed in the desired electrosynthetic transformation. A simpler method, which does not require switching of the polarity, places the electrodes in a caustic soda solution, followed by constant current electrolysis in the activation solution without polarity switching [52]. When immersing the electrodes in the caustic soda, the formation of flocculants is observed, which is likely Ni(OH)_2_. This facilitates the formation of NiOOH in the subsequent electrolysis step, which leads to the activation of the nickel foam electrode.

### 3.2 The Solubility of Substrates

Because NiOOH electrodes require an alkaline aqueous electrolyte, the reaction medium influences the solubility of substrates. Often, higher temperatures seem beneficial for transformations of more hydrophobic substrates, such as longer‐chain alcohols [50]. To increase solubility, a common trick is to add up to 30% *tert*‐butanol to the reaction mixture. An alternative is to employ a biphasic system for electrolysis [28]. Another approach is the addition of amines, but this alters the electrode surface chemically [53]. In addition, several attempts have been developed to employ surfactants in the electrolysis, which leads to the formation of an emulsion [54]. Such electrolytic systems are an example of electrochemistry under confinement, as they alter the underlying physical processes, such as mass transport, due to the formation of micelles [55]. However, these surfactants can be problematic for phase separation in the downstream processing. It should be mentioned that some limitations are still left to be solved. While several concepts exist to increase the solubility of lipophilic substrates in aqueous alkaline media, these methods are not sufficient yet to allow for electrolysis of highly lipophilic substrates, such as betulin [56].

**SCHEME 3:**
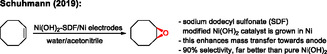
Electrochemical epoxidation of cyclooctene using water via a sodium dodecyl sulfonate‐modified nickel foam anode [54].

In 2019, the Schuhmann group demonstrated an electrocatalytic epoxidation of cyclooctene on surface‐modified nickel foam electrodes using water as a source of oxygen (Scheme 3) [54]. A key development of this work is to increase the lipophilicity of the electrode surface by using a sodium dodecyl sulfonate‐modified nickel hydroxide catalyst that is grown on nickel foam. The use of water for epoxidation of alkenes is a sustainable alternative to conventional epoxidation reagents [57]. This improved lipophilicity of the electrode surface facilitates mass transfer of cyclooctene toward the anode [55], which results in increased conversion and selectivity compared with pure Ni(OH)_2_. The work by Schuhmann was motivated by the necessity to find a matching counter reaction for the hydrogen evolution reaction (HER) [19, 20], which is utilized for hydrogen production as an energy carrier. It aims to offer alternatives to the oxygen evolution reaction (OER) at the anode, which is not beneficial due to the usually high overpotential. Also, O_2_ is a comparably low‐value product, since Earth already possesses an oxygen‐containing atmosphere, making air the most inexpensive oxidant of choice on an industrial scale. Compared to that, epoxides are high‐value products that are required in large amounts for various applications such as epoxy resins [58]. While his work is an interesting proof of concept, further work is required to bring this into practical application. Nonetheless, the use of surfactants to form emulsions is an innovative concept that deserves further exploration.

### 3.3 Oxidation of Primary Alcohols to Carboxylic Acids

**SCHEME 4:**
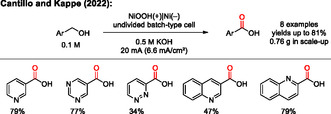
NiOOH‐mediated anodic oxidation of benzylic alcohols [60].

The electrochemical oxidation of benzoyl alcohols to their carboxylic acids using nickel foam anodes was reported by Schäfer in 1979 [59]. Inspired by this, Kappe and Cantillo in 2022 reported a method for the mediated anodic oxidation of primary alcohols to carboxylic acids using NiOOH anodes (Scheme 4) [60]. To optimize the anode pretreatment, the anodic oxidation of cyclohexanol to cyclohexanone using nickel electrodes in an an aqueous alkaline solution in an undivided cell was chosen [60]. While all nickel anodes with pretreatment gave satisfying yields, only some of the nickel anodes without pretreatment performed in an acceptable range and only when a higher amount of charge was applied. Without any pretreatment, a higher amount of applied charge is also required, and still, more inconsistent results were observed. Reaction optimization was carried out using the anodic oxidation of nicotinyl alcohol to nicotinic acid [60]. It was observed that due to the neutralization reaction between the carboxylic acid product and the alkaline media, increasing the concentration of KOH was required. The optimized reaction conditions were then applied to various nitrogen heterocycle‐containing primary alcohols. The group also transferred this protocol into a flow. The challenge of hydrogen gas evolution in single‐pass flow, which results in filling of the nickel foam with gas, was overcome by adopting a cascade protocol, whereby the reaction solution is passed through the cell in single‐pass mode, three times consecutively.

### 3.4 Oxidation of Enolizable Cyclohexanols

**SCHEME 5:**
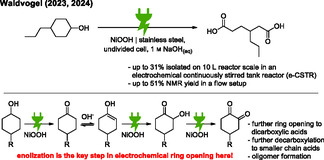
Anodic oxidation of 4‐alkylcyclohexanols to 3‐methyl adipic acids. Enolizable ketones are the key intermediates formed by anodic oxidation of cyclohexanols [52].

In 2020, the Waldvogel Group reported an electrochemical oxidation of lignin‐derived alkylated cyclohexanols to alkylated adipic acids using nickel foam anodes in caustic soda (Scheme 5) [61]. This approach developed a simple protocol using NiOOH foam anodes in caustic soda on both batch‐type and flow electrolysis cells. Importantly, no other additive than caustic soda was required. The valorization of lignin is important because it is a sidestream of the pulping process, and large amounts of this biopolymer accumulate as a byproduct. Various alkylated cyclohexanols can be obtained from the reductive depolymerization of lignin. The adipic acids obtained via anodic oxidation of these cyclohexanols could serve as important building blocks for polymers such as polyamides or polyesters [62]. For reaction optimization, 4‐methylcyclohexanol was employed as the test molecule. Notably, alkylated glutaric acid derivatives were obtained as side products as well, and because these were not formed by oxidation of the adipic acids, it was concluded that these must have formed via the partial oxidation of the cyclohexanone ring in the early stage of the conversion. To activate the nickel electrodes, a protocol from Schäfer et al. was followed, which employed a buffered NiSO_4_/NaOAc/NaOH solution for anodic pretreatment. The scale‐up of this reaction was carried out in a flow reactor using a single‐pass mode to enhance the surface‐to‐volume ratio and mixing. The advantage of cyclic flow is the removal of hydrogen gas bubbles from the flow electrolysis cell, which would block the electrode surface and decrease the conversion.

In 2023, the Waldvogel group reported an electrochemical synthesis of 3‐alkyladipic acids via anodic oxidation of 4‐alkylcyclohexanols in a continuously stirred tank reactor [52]. One major discovery is that enolization of the ketones plays a major role in the reaction pathways that occur. These processes also use a NiOOH anode, which regenerates in basic aqueous media upon electrolysis. The use of 4‐alkylcyclohexanols is interesting because they can be obtained by degradation and hydrogenation of lignin, which is a major component of the biomass of trees [63]. The obtained dicarboxylic acids are valuable starting materials for various applications, such as the synthesis of polyamides [64]. The reaction conditions were optimized using Design of Experiments (DoE) [65]. This work was extended shortly after into a scalable process in a flow reactor [66, 67]. In this follow‐up work, a novel activation process for nickel anodes was developed using caustic soda.

### 3.5 Electrochemical Oxidative Degradation of Lignin

**SCHEME 6:**
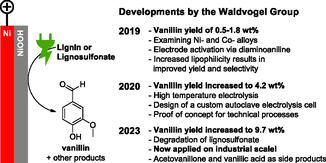
Electrochemical oxidative degradation of lignin to value‐added products [70, 71, 72, 73, 74, 75, 76].

Lignin is one of the most abundant biopolymers on the planet, which originates from plants [68].Its electrochemical oxidative degradation yields various interesting building blocks such as vanillin, acetovanillone, or vanillic acid. A major advantage of lignin valorization is that it does not compete with nutritional feedstocks. In 2019, the Waldvogel Group utilized metal‐based alloys to increase the performance of NiOOH anodes for lignin degradation (Scheme 6) [66, 67]. Cobalt alloys perform with better yield but suffer from severe corrosion. The repeated use of nickel foam electrodes for the anodic degradation exhibits an increase in the yield of vanillin with operational time, which indicates surface modification of the nickel electrodes. In this report, it was found that diaminotoluene was a major component of the modified electrode surface in the activation process. This improves the lipophilicity of the electrode surface, which affects mass transfer and hence conversion and yield. Notably, the yield reached a plateau after several electrolysis runs, which demonstrates the stability of the layer that formed on the electrode. Also, the selectivity of the degradation could be improved toward the formation of vanillin. Several nickel‐ and cobalt‐based alloys were tested for the anodic degradation of Kraft lignin, which is a waste stream originating from the pulping process. Cobalt‐based materials usually suffer from corrosion and decreased electrocatalytic activity compared with nickel‐based materials.

In 2020, the Waldvogel Group further developed the electrochemical degradation of lignin by designing high‐temperature electrolysis in an autoclave [69]. In this report, the selectivity for vanillin product formation was optimized using a linear screening approach based on high‐temperature electrolysis. This report also investigated the influence of current density, applied charge, and electrode morphology. This led to an improved yield for vanillin. This process was demonstrated for various commercially available types of Kraft lignin. As expected from the previous report, the temperature plays a crucial role in the yield and selectivity of this process. At around 160°C, planar nickel electrodes and nickel have similar performance since faster kinetics overrule the higher surface material.

In 2023, the Waldvogel Group reported the selective electrochemical degradation of technical lignosulfonate [70]. In addition to vanillin, this protocol also yields acetovanillone or vanillic acid as side products in lower yields. A key advancement in this work is the separation of the electrolysis step from the thermolysis, which is a one‐pot two‐step degradation overall. This increased the yield of vanillin to 9.7 wt% relative to the dry mass of used lignosulfonate. All steps were carried out in an undivided pressurized electrolysis cell (an autoclave), which allows for safe operation in aqueous media at temperatures above 100°C. It should be noted that the alkaline aqueous media not only lead to the formation of NiOOH on the anode, but they also increase the solubility of the lignosulfonate. Nickel was found to give the highest yields, while nickel alloys and various other electrode materials were inferior, as corrosion was observed in some cases. Notably, further research was carried out by the Waldvogel group in the area of electrochemical valorization of lignin [71], also using other electrode materials such as boron‐doped diamond (BDD) [72, 73, 74, 75, 76].

### 3.6 Other Anodic Oxidations of Biomass

**SCHEME 7:**
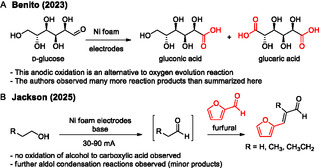
Electrochemical oxidative degradation of lignin to value‐added products [77, 78].

In 2023, the group of Benito reported an anodic oxidation of D‐glucose in alkaline media using nickel foam electrodes (Scheme 7A) [77]. This anodic oxidation was also developed to be an alternative to the oxygen evolution reaction (OER), which commonly produces oxygen as a comparably low‐value product in water electrolysis. The reaction was carried out under potentiostatic conditions, which gave a broad variety of further products. The authors demonstrated both the anodic oxidation of glucose to gluconic acid and the consecutive oxidation of glucose at C6 to glucaric acid. Fructose underwent C—C bond cleavage to yield arabinose, glyceric acid, and formic acid. The stability of the NiOOH electrocatalyst was demonstrated by reusing it in several cycles, and the product distribution was investigated with respect to reaction parameters, and several side reactions were observed.

The Jackson group reported an electrochemical method for the oxidative aldol condensation of 1‐propanol and ethanol with furfural using nickel foam electrodes in alkaline media (Scheme 7B) [78]. A high Faradaic efficiency was achieved, and the authors demonstrated the stability of the nickel foam electrodes. This methodology allows for the electrochemical in situ generation of aldehydes, which then react with alcohols in aldol condensation as followup reactions, avoiding isolation of the aldehyde entirely.

## 4 Nickel Foam as Cathode Material

Nonmodified nickel foam is a surprisingly versatile cathode material for reductive electro‐organic synthesis. Although nickel has a strong tendency to promote the hydrogen evolution reaction, it can still be loaded with enough adsorbed hydrogen to drive hydrogenation reactions by its intrinsic catalytic features. Moreover, under aprotic conditions, nickel foam can reduce organic substrates, CO_2_, or metal salts, enabling carboxylations as well as metal‐ and radical‐mediated cross‐coupling reactions. Owing to its large surface area, this material provides a useful platform for reductive transformations at a relatively high current density, enhancing productivity while maintaining good chemoselectivity.

### 4.1 Hydrogenation Reactions

**SCHEME 8:**
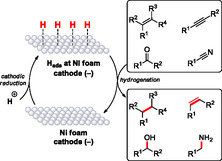
Commonly proposed mechanism for hydrogenation of organic substrates at nickel foam.

Nickel foams serve as bulk, cost‐effective catalysts for proton reduction, which results in the formation of hydrogen adsorbed at the nickel surface (*H*_ads_). While much research has focused on optimizing conditions to enhance the hydrogen evolution reaction (HER) from this intermediate [41], its application in the hydrogenation of organic substrates (Scheme 8) received comparably less attention. This is surprising considering the widespread use of Raney nickel in classical chemistry, which is mechanistically based on the same *H*_ads_ intermediate. Notably, hydrogen‐loaded Raney nickel is pyrophoric in particular when getting dry, whereas nickel foams are much safer and not pyrophoric. Under electrochemical conditions, handling of pressurized hydrogen is completely circumvented, and importantly, new opportunities to achieve better selectivity arise due to generally milder reaction conditions. Moreover, electrochemical conditions permit the use of strongly acidic media, which is beneficial for certain classes of substrates (vide infra). However, this would not be possible under conventional conditions. To prevent dissolution (corrosion) of nickel, the foam has to stay electrically polarized.

#### 4.1.1 Hydrogenation of C—C Multiple Bonds

**SCHEME 9:**
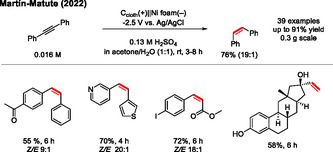
Selected synthetic scope examples of partial hydrogenation on nickel foam under acidic conditions [46].

In 2022, Martín‐Matute reported a selective hydrogenation method for partial hydrogenation of alkynes to the corresponding Z‐alkenes on nickel foam. This electrolysis is based on using a well‐conducting aqueous sulfuric acid‐based electrolyte in a divided cell equipped with a glass frit separator under potentiostatic conditions. The authors demonstrated good to excellent yields of up to 91% for a variety of terminal or nonterminal alkynes and high Z/E ratios of up to 20:1 (Scheme 9) [46]. The conditions impressively tolerate different easily reducible molecular entities, such as carbonyls; haloarenes, including aryl iodides; as well as nitrogen‐ and sulfur‐containing heterocycles. This transformation has also been reported earlier by Zhang using Se‐vacancy‐enriched Ni_0.85_Se nanowires on nickel foam substrate [33]. The advanced material enabled authors to achieve higher yields and selectivity than bare nickel foam, presumably due to a different reaction mechanism based on direct alkyne reduction.

**SCHEME 10:**
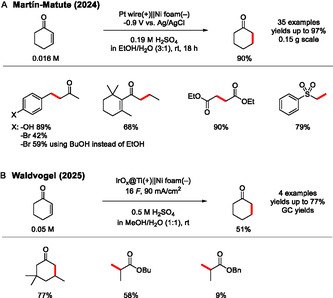
(A) Hydrogenation of enones under potentiostatic and (B) galvanostatic conditions [79, 80].

In combination with deuterated solvents and additives, both methods for hydrogenation of alkynes become powerful deuteration platforms, allowing for more than 95% deuterium incorporation and high yields. Later, in 2024, the Martín‐Matute group reported a method for the hydrogenation of enones (Scheme 10A) [79]. The potentiostatic protocol achieves selective C = C hydrogenation over C = O reduction, using a glass frit separator in a divided cell with an aqueous sulfuric acid‐based electrolyzer and a Pt wire counter electrode. The method tolerates a broad range of sensitive functional groups, including aryl bromides, sulfonates, and esters. In certain cases, replacing EtOH with BuOH led to significant improvements in yield. Interestingly, the sustainability analysis in this work found that sourcing a stable anode material represents a major environmental "hotspot."

Soon after, the Waldvogel group published a method for hydrogenation of enones under galvanostatic conditions, employing a Nafion cation‐exchange membrane and a dimensionally stable iridium oxide‐coated anode (Scheme 10B) [80]. These features—galvanostatic operation and the use of a membrane as a separator—are highly advantageous for scale‐up in flow‐type electrolyzers. The authors achieved good yields of the desired unsaturated products at a relatively high geometric current density 90 mA/cm^2^ using sulfuric acid in a MeOH–H_2_O solvent system. The aim of using high electrical currents was to make the method more appealing for foreseeable industrial applications. The authors observed diminished performance of the developed hydrogenation system with more lipophilic substrates due to their lower solubility.

#### 4.1.2 | Hydrogenation of C—O Multiple Bonds

**SCHEME 11:**
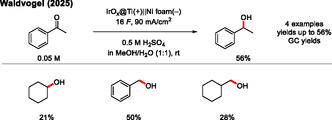
Hydrogenation of carbonyl compounds to the corresponding alcohols [80].

The system for reduction of enones developed by the Waldvogel group can also be applied to reduction of C = O bonds with substrates that don’t contain any C = C bonds (Scheme 11) [80]. Using the same electrolysis conditions with high current density, the authors managed to obtain good yields of alcohols from the corresponding ketones or aldehydes as substrates.

Despite sometimes impressive yield and selectivity in the cathodic transformations presented so far (Schemes 9–11), a major problem of these systems lies in using water alongside alcohols in anolyte. This inevitably leads to the formation of nonnegligible amounts of highly toxic aldehydes (e.g., formaldehyde, acetaldehyde) at the anode. The 2‐electron alcohol oxidation is kinetically and thermodynamically favored over complex 4‐electron water oxidation [81].

#### 4.1.3 | Hydrogenation of C—N Multiple Bonds

**SCHEME 12:**
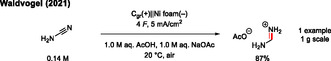
Hydrogenation of cyanamide to formamidine acetate [82].

In 2021, the Waldvogel group reported hydrogenation of cyanamide to formamidine acetate—an important and versatile building block in synthetic organic chemistry (Scheme 12) [82]. Electrolysis proceeded in a divided batch‐type cell using a glass frit separator and aqueous sodium acetate buffer‐based electrolyte, giving a high yield of 87%. Apart from conductivity, acetate ions have an additional role as a species being oxidized in the anodic counter reaction [19, 20, 83]. Interestingly, under the developed galvanostatic conditions, the nickel plate electrode performed only slightly worse than nickel foam, giving 79% yield. Upon switching to a different reactor setup, a 12 cm^2^ flow‐type cell using Nafion proton exchange membrane, the electrolysis yields dropped. After reoptimizing the reaction parameters in flow, the authors were able to achieve 54% yield on a gram scale, importantly, without using sodium acetate salt as a supporting electrolyte. The formed product served as a supporting electrolyte and could start crystallizing in the reservoir. Nickel foam got stained in these experiments but, interestingly, still retained its activity. The counter reaction in flow electrolysis was the oxygen evolution reaction.

**SCHEME 13:**
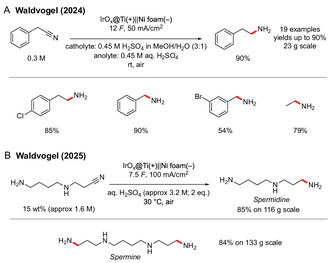
General hydrogenation of nitriles (A) and a specific case study for the preparation of two technically important polyamines (B) [84, 85].

In 2024, Waldvogel reported a selective hydrogenation of organic nitriles to obtain primary amines (Scheme 13A) [84]. In the case of nitriles, the hydrogenation is a two‐step process proceeding via the formation of a reactive aldimine intermediate (R‐CH = NH), which is prone to side reactions such as attack of nucleophilic amine, resulting in the formation of undesired over‐alkylated by‐products. Under strongly acidic conditions, these side reactions are suppressed because the formed amines get immediately protonated and thereby lose nucleophilicity. Utilizing this strategy, conditions were developed for a divided batch‐type cell using aq. sulfuric acid electrolyte and Nafion membrane as a separator. The reported protocol is applicable to different organic nitriles, as the catholyte consisted of water and methanol as co‐solvents (3:1), ensuring good solubility of organic compounds, while the anolyte contained only water to avoid the formation of toxic formaldehyde. The electrolysis was optimized to yield 90% of the medicinally relevant compound phenylethylamine at a relatively high geometric current density of 50 mA/cm^2^ in a batch‐type cell. The transformation works for different benzonitriles, benzylnitriles, and simple aliphatic nitrile derivatives, tolerating some easily reducible substituents like bromine substituents. Substrates with sterically less accessible nitrile groups react more slowly and therefore give lower conversions and yields under the standard conditions. The authors optimized the reaction in two flow‐type electrolyzers with geometric foam electrode sizes of 12 and 48 cm^2^. Interestingly, they found that the conditions from batch‐type cells are also optimal in both flow cells, giving the same reaction yield as in batch, that is, 90%. Using the larger cell, the authors managed to prepare 22 g of the phenylethylamine, corresponding to a productivity of 20 g/day. The large‐scale experiment and a series of consecutive electrolytic runs demonstrated good durability and reusability of the electrolyzer components: Ni foam cathode, IrO_*x*_@Ti anode, Nafion, and tubing. Building on these findings, subsequent work by the Waldvogel group in 2025 extended this approach to the synthesis of technically relevant polyamines spermidine and spermine (Scheme 13B) [85]. These molecules are notoriously difficult to prepare by classic hydrogenation because of the high tendency of aldimine intermediates to undergo an intramolecular cyclization reaction with tethered amine groups, leading to a complex reaction mixture of products. The authors developed an attractive electrochemical alternative using nickel foam cathodes in aqueous sulfuric acid electrolyte, which enables the selective formation of the desired primary amines. With a strong emphasis on practicality, the concentration of the substrate was increased to 15 wt% (ca. 1.6 M), and in combination with a high geometric current density of 100 mA/cm^2^, approximately 85% yield for both products was achieved in short electrolysis times. The high product concentration allowed simple precipitation‐based isolation and purification protocols, avoiding energy‐intensive distillation or chromatographic purification. Despite the acidic electrolyte and high ability of polyamines to chelate metal ions, the final electrolysis mixtures contained only approximately 15 ppm of Ni and the isolated products approximately 5 ppm. The nickel is not present in higher amounts due to the applied negative cathodic potential, highlighting a clear advantage of electrochemistry over classical hydrogenations, which would yield a severely metal‐contaminated product.

**SCHEME 14:**
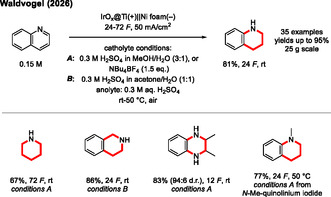
Hydrogenation of different classes of N‐heterocycles [86].

In 2026, Waldvogel reported a system using Ni foam and aqueous sulfuric acid electrolyte for hydrogenation of nitrogen heterocycles (Scheme 14) [86]. The system is compatible with a wide range of differently functionalized six‐membered nitrogen heterocycles: pyridines, quinolines, isoquinolines, quinoxalines, phenanthroline, and their cationic salts. The key for successful dearomatization is the charged nature of the N‐heterocyclic scaffold achieved either via protonation or alkylation. To achieve high yields of hydrogenation of all these different classes of heterocycles, the authors established two sets of conditions; in one case, the catholyte consisted of MeOH‐water (3:1), and in the other case, it consisted of acetone‐water (1:1) with 1.5 additional equivalents of NBu_4_BF_4_ supporting electrolyte. For the compounds containing multiple protonatable nitrogen atoms, trifluoromethanesulfonic acid was used instead of sulfuric acid for solubility reasons. Extensive mechanistic investigations using a combination of cyclic and RDE linear sweep voltammetry suggest two plausible routes based on the substrate's redox properties: hydrogenation by chemisorbed hydrogen (*H*_ads_) or by direct cathodic reduction of substrate. Similarly, as previously developed systems, these conditions are readily scalable in flow setup to at least 20 g scale.

### 4.2 Carboxylation Reactions

**SCHEME 15:**
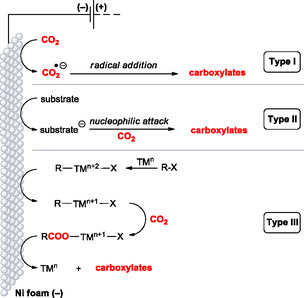
Different possible carboxylation mechanisms on nickel foam cathode. TM: transition metal [87].

Ni foam found applications in carboxylation reactions as well. The reaction mechanisms of the transformations differ and can be divided into three categories: types I, II, and III (Scheme 15) [87]. The operating mechanism depends on the redox properties of the substrates and the use of catalytically active mediators like transition metals.

#### 4.2.1 | Carboxylation of Multiple Bonds

**SCHEME 16:**
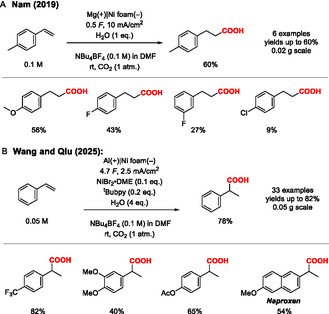
β‐Hydrocarboxylation of styrenes with water as a proton source following type I and II mechanisms (A) [88] and β‐hydrocarboxylation of styrenes following type III mechanism (B) [89]. DME: 1,2‐dimethoxyethane.

In 2019, Nam reported a method for hydrocarboxylation of styrenes (Scheme 16A) [88]. The reaction is performed in an undivided cell using aprotic CO_2_‐saturated dimethylformamide (DMF) as a solvent and NBu_4_BF_4_ (0.1 M) as a supporting electrolyte in combination with a magnesium sacrificial anode. Under completely anhydrous conditions, the authors reported the formation of dicarboxylic acids, whereas upon the addition of a carefully selected proton donor, the reaction outcome changes. After screening different proton sources, they noticed that the addition of one equivalent of water completely suppresses the formation of diacids, giving up to 60% yield of β‐hydrocarboxylation products. The reaction yields are higher with electron‐donating substituents, whereas electron‐withdrawing groups, including halogens, are not well tolerated. The results of voltammetric studies suggest that both the type I and II mechanisms are possible under the reaction conditions.

In 2025, Wang and Qiu reported Ni‐catalyzed carboxylation of styrenes following a type III mechanism [89]. Despite similarities in reaction conditions with previously reported work of Nam, the addition of a nickel complex, in this case, makes a difference in the regioselectivity of the obtained products, now yielding α‐hydrocarboxylation products (Scheme 16B). These products were obtained in high yields with observed good functional group tolerance and low sensitivity of yields to the electron properties of styrene substrates. Importantly, using this protocol, the authors prepared a few examples of drug molecules, including racemic form of Naproxen.

**SCHEME 17:**
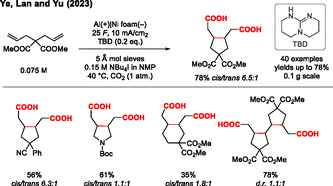
Radical dicarboxylation of nonactivated dienes with CO_2_ [90]. NMP: *N*‐methylpyrrolidone, TBD: 1,5,7‐triazabicyclo[4.4.0]dec‐5‐ene.

In 2023, Ye, Lan, and Yu reported a method to afford value‐added dicarboxylic acids from nonactivated 1, n‐dienes (*n* > 3) (Scheme 17) [90]. The transformation was performed in an undivided cell, using a catalytic amount of nitrogen base 1,5,7‐triaza‐bicyclo[4.4.0]dec‐5‐ene. (TBD) and NBu_4_I as supporting electrolyte in dry NMP at 40°C under CO_2_ atmosphere. The authors selected 1,6‐dienes, which are substituted at position 4 and are thus very likely to undergo radical 5‐exo‐trig cyclization due to the Thorpe–Ingold effect [91]. Using this strategy, the authors achieved up to 78% high yields of the desired dicarboxylic acid. The dicarboxylative cyclization works well, giving 5‐ or 6‐membered dicarboxylates, and tolerates a variety of functional groups, including ethers, protected amides, nitriles, and esters. Depending on the substituents, the products were obtained as cis/trans isomers or diastereoisomers in ratios ranging from 1:1‐8.4:1 (cis preferred). The authors also showed an impressive example of a triene substrate, which cyclizes in a radical cascade in two 5‐membered ring dicarboxylic acids in high 78% yield. The obtained products could serve as building blocks in polymer chemistry. The method does not seem to tolerate halo substituents. Under the reaction conditions, SET reduction of CO_2_ is way easier than SET reduction of diene, and therefore, the described reaction likely proceeds via a type I mechanism.

#### 4.2.2 | Carboxylation of Halides

**SCHEME 18:**
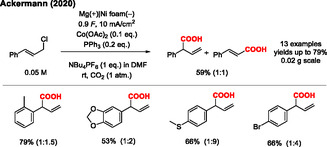
Cobalt‐catalyzed carboxylation of cinnamyl chlorides [92, 93].

In 2020, Ackermann reported a method for carboxylation of cinnamyl chlorides using a cobalt catalyst to obtain carboxylic acids from cinnamyl chlorides (Scheme 18) [92, 93]. The reaction was performed in an undivided cell using a simple system based on cobalt acetate and triphenylphosphine in catalytic quantities and NBu_4_PF_6_ as supporting electrolyte in DMF under 1 atm of CO_2_. The method tolerates some easily reducible moieties like aryl bromides and acid‐labile ethers, as well as sulfur‐containing methyl sulfide groups. The products were obtained as mixtures of regioisomers due to the rearrangement of *η* ^1^‐allyl‐cobalt(III) intermediates.

**SCHEME 19:**
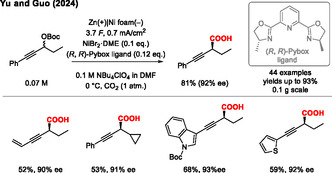
Asymmetric nickel‐catalyzed carboxylation of propargylic carbonates [94]. DME: 1,2‐dimethoxyethane; Pybox: pyridine bis(oxazoline).

Later in 2024, Yu and Guo presented a system for the synthesis of enantioenriched propargylic carboxylic acids from racemic propargylic carbonates and CO_2_ (Scheme 19) [94]. The system is based on the nickel bromide precatalyst in combination with the tridentate PyBOX ligand, using NBu_4_ClO_4_ as a supporting electrolyte in DMF under 1 atm of CO_2_ at 0°C. The authors presented an extensive synthetic scope, showing high generality of transformation and giving an impressive high enantiomeric excess of up to 98% and mostly very high yields. Different acid‐sensitive substituents, multiple bonds, strained cycles, and chlorides are all well tolerated. To show the importance of the synthesized motif, the authors successfully utilized the products in the asymmetric total synthesis of (*S*)‐arundic acid, (*R*)‐PIA, (*S*)‐chizhine D, (*S*)‐cochlearin G, and (*S*,*S*)‐alexidine.

**SCHEME 20:**
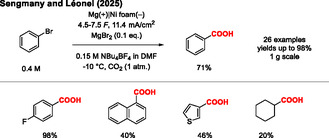
Carboxylation of aryl bromides [95].

In 2025, Sengmany and Léonel reported an electrochemical system for carboxylation of aryl bromides (Scheme 20) [95]. The authors noted that the system is quite sensitive to protons coming from water in DMF. To alleviate this issue, they have run pre‐electrolysis with dibromoethane before each electrolysis. The preparative electrolyzes were performed at ‐10°C in DMF using NBu_4_BF_4_ as an electrolyte in combination with a catalytic amount of magnesium bromide. The reaction conditions gave a whole range of yields from poor to almost quantitative yields. As for the mechanism, the authors propose in situ generation of organomagnesium bromide that reacts with CO_2_. The fact that an overstoichiometric amount of magnesium (sacrificial anode) is required brings up an important point of criticism. Formation of Grignard reagents from magnesium fillings and aryl bromide is a well‐established classic reaction that does not require any applied current to proceed. Likewise, quenching the Grignard reagent with the CO_2_ step does not require any electricity [96]. The advantages of using relatively complicated electrochemistry over classic thermal conditions are not immediately clear in this case.

### 4.3 Ni‐catalyzed Cross‐coupling Reactions

**SCHEME 21:**
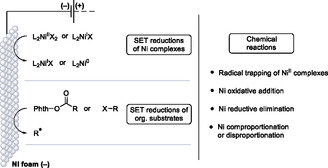
Mechanisms of nickel‐catalyzed cross‐couplings on nickel foam cathode [97, 98, 99].

Nickel foam and nickel plate are very commonly used electrodes in combination with nickel complexes to perform different cross‐coupling reactions. In these systems, reactions happening at the electrodes are SET reductions of nickel complexes to their lower oxidation state and SET reductions of easily reducible organic compounds such as phthalimide esters or some acyl or alkyl halides, leading to the formation of organic radicals (Scheme 21). These electrochemical steps work in synergy with classical chemistry happening in the solutions, which consist of fundamental reactions in organometallic chemistry: oxidative additions, reductive elimination, radical trapping by nickel complexes, and different comproportionation or disproportionation reactions [97, 98, 99]. Mechanisms are different and depend on coupling partners and the tuned properties of nickel complexes. In general, cross‐coupling reactions can be divided into net reductive cross‐electrophile couplings and redox‐neutral coupling of a nucleophile and an electrophile (e.g., Buchwald–Hartwig amination). The difference is that the former class requires some type of sacrificial reducing agent (typically a sacrificial anode), and the latter class does not.

Due to a very large number of possibilities of coupling partners, many electrochemical conditions have been developed to date (50+). Very recently, multiple high‐quality reviews [98, 99, 100, 101] made a heroic effort of summarizing them along with relevant discussions about possible mechanisms. At the current stage, this technology is well developed and applicable to simple small organic molecules as well as more complex peptides and nucleosides [102, 103]. Therefore, we present only a limited selection of works that demonstrate scale‐up beyond 5 g or report practically relevant strategies for improving yields and productivity. This goes in line with our belief that nickel foam is a great electrode material with high potential for scale‐up applications. Some of the presented reactions have been shown to work on glassy carbon as well; however, using nickel foam typically enables higher current density due to the higher electrode surface.

**SCHEME 22:**
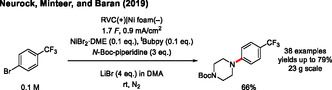
Electrochemically driven nickel‐catalyzed amination of aryl halides [103].

In 2019, Neurock, Minteer, and Baran reported an electrochemical method for Buchwald‐Hartwig‐type amination using nickel salts (Scheme 22) [103]. The method relies on the use of a simple bipyridyl ligand and nickel precatalyst in an undivided cell using LiBr as a supporting electrolyte in dimethylacetamide (DMA). The reaction conditions using 3‐fold excess of nucleophile relative to the aryl bromide or chloride electrophile are very general, as they can be applied to a variety of heterocycles, as well as amino acids, peptides, and nucleosides. In the case of amino acids, the authors reported a minor loss of enantiomeric excess. The reaction was successfully scaled up to >20 g in a batch‐type reactor consisting of a large thin‐layer chromatography (TLC) chamber in an argon bag to ensure an inert atmosphere.

**SCHEME 23:**
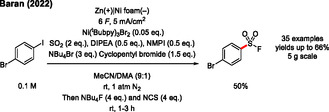
Sulfonylation of aryl halides with SO_2_ gas stock solution [104].

Later in 2022, Baran reported an example of nickel‐catalyzed sulfonylation of aryl halides with SO_2_ stock solutions (Scheme 23) [104]. The electrolysis is performed in a simple, undivided cell setup, using a sterically hindered tertiary amine DIPEA additive, which forms adducts with SO_2_ [105]. These in situ‐formed adducts are presumably less prone to undergo aggregation upon SO_2_ reduction and cause catalyst poisoning. The authors noted that 0.5 eq. NMPI beneficially affects the reaction yields and functional group tolerance. As such, the method is applicable to a wide variety of aryl bromides and iodides. The authors made an interesting observation of an ‘induction’ period before the start of the product formation. They attributed the first 2 F of charge to the reduction of the amine‐SO_2_ complex. After the buildup of the SO_2_ radical anion, the actual sulfonylation of aryl compounds starts. The reaction was successfully transferred in flow and scaled up to obtain 5 g of the desired fluorinated sulfonylated product.

**SCHEME 24:**
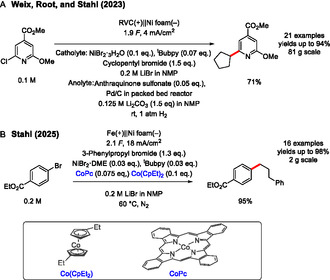
(A) Electrochemical cross‐electrophile coupling with hydrogen gas as a sacrificial reductant under anhydrous conditions [106] and (B) Cobalt‐mediated electrochemical nickel‐catalyzed cross‐electrophile coupling (XEC) [108].

In 2023, Weix, Root, and Stahl reported a cross‐electrophile coupling with a novel design of the anodic compartment, effectively using hydrogen gas as a sacrificial reducing agent (Scheme 24A) [106]. The setup used in this system was a divided cell using Nafion as a separator. The anolyte was continuously circulated through a Pd‐bed reactor that, in combination with hydrogen gas, reduced sulfonated anthraquinone mediator to its diol form. Oxidation of the diol at the anode served as a counter reaction, releasing only proton flux, thus effectively mediating H_2_ oxidation. A similar idea of oxidizing hydrogen has also been realized with deposited platinum on membrane electrode assembly (MEA) in the context of hydrogenations [107]. By using the anthraquinone‐mediated system, the authors successfully applied the conditions to a variety of aryl and alkyl bromides, achieving up to 94% yields. Additional validation of this approach was demonstrated by a scaleup in a recirculating flow setup used for preparation of a pharmaceutical intermediate for preparation of the active pharmaceutical ingredient (API) Cenerimod on an 80‐gram scale by coupling chloropyrdine with a slight 1.5 eq. excess of cyclopentyl bromide.

Later, in 2025, Stahl published a modification of nickel‐catalyzed C(sp^2^)—C(sp^3^) bond‐forming cross‐electrophile coupling addressing an important issue of slow rates and therefore low current densities (<4 mA/cm^2^) typically required in such systems. Their solution relies on the use of homogeneous electron‐transfer (ET) mediators cobalt phthalocyanine and cobaltocene (Scheme 24B) [108]. Using these two additives, the authors were able to achieve higher selectivity for the desired product at higher current densities (18 mA/cm^2^), thereby significantly increasing the productivity and attractiveness of Ni‐catalyzed cross‐electrophile coupling strategies for practical application.

**SCHEME 25:**
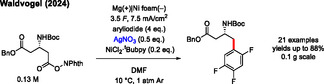
Silver nanoparticle‐catalyzed nickel cross‐electrophile coupling [102].

As a next practically relevant highlight in Ni‐catalyzed cross‐couplings, we want to highlight a strategy introduced by Engle and Baran on RVC and the Waldvogel group on Ni foam cathode in 2024 [102, 109]. In this approach, silver salt is added to the reaction mixture, which leads to electrodepositing of silver nanoparticles during electrolysis. The formed nanoparticles significantly lower the overpotential at the electrode and generally lead to a significant increase in the desired cross‐coupling product yields. The Waldvogel group utilized this approach for the preparation of a library of Sitagliptin precursors (Scheme 25) from a phthalimide ester radical precursor and different aryl iodide electrophiles [102].

### 4.4 Cocatalyzed Crosscoupling Reactions

Similar to nickel salts, cobalt salts have also been employed in combination with nickel foam electrodes to enable a range of electrochemically driven cross‐coupling and radical‐mediated transformations. Electrochemical events at the electrode typically involve the reduction of cobalt complexes to lower oxidation states and, in some cases, the direct SET reduction of readily reducible organic substrates. These electrochemical steps operate in concert with classical solution‐phase organometallic chemistry, including oxidative addition, radical capture by cobalt species, bond‐forming reductive elimination, and various comproportionation or disproportionation processes [110, 111, 112]. As in the case of nickel‐based systems, the precise mechanistic details vary depending on the nature of the coupling partners, the ligand environment around cobalt, and the applied electrochemical conditions. Two examples of Co‐mediated reactions were already presented in the previous chapters: Co‐mediated carboxylation in Scheme 18 and Co‐mediated Ni‐cross‐coupling in Scheme 24B.

**SCHEME 26:**
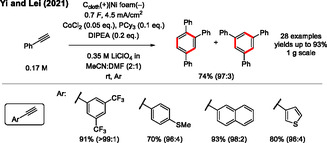
Cocatalyzed cyclotrimerization of alkynes [113]. The numbers in the brackets represent the ratio between the major 1:2:4 and minor 1:3:5 substituted isomer.

In 2021, Yi and Lei reported an electrochemical cobalt‐catalyzed [2 + 2 + 2] cyclotrimerization of alkynes to obtain different 1,2,4‐trisubstituted and hexasubstituted benzenes (Scheme 26) [113]. The reaction proceeds under galvanostatic conditions in an undivided cell, relying on the use of stable anodes in combination with CoCl_2_ and phosphine ligand system. Mechanistic proposal is based on the cathodic reduction of cobalt (II) catalyst to cobalt (0) species, which then coordinates with alkynes and, upon oxidative addition, forms a metallacyclic intermediate. DIPEA is interestingly added in catalytic amounts, and its proposed role is mediation of the electron transfer between the metallacyclic intermediate and the carbon anode. The method can be applied to a variety of substrates, including heterocycles and completely aliphatic substrates, which can undergo intramolecular cyclization.

**SCHEME 27:**
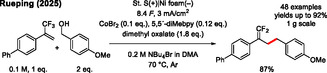
Cofacilitated deoxygenative defluorinative radical crosscoupling [114].

In 2025, Rueping reported a strategy for deoxygenative defluorinative radical coupling of benzyl alcohols and α‐CF_3‐_substituted styrenes (Scheme 27) [114]. The system uses dimethyloxalate as an additive, which, under electrolysis conditions, reacts with benzyl alcohols to form esters. The benzyl radical formation and deoxygenation steps happen upon the reduction of the oxalate ester. Subsequent radical trapping by styrene and cobalt species and reduction at the nickel electrode leads to anion formation in the α position to the CF_3_ group, which undergoes fluoride elimination and formation of the desired gem‐difluoro alkenes. The reaction, interestingly, also works similarly well with NiCl_2_ salt instead of CoBr_2_.

### 4.5 Miscellaneous Recent Developments

**SCHEME 28:**
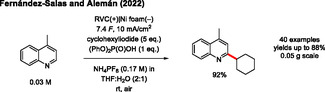
Electrochemical Minisci alkylation of N‐heteroarenes with alkyl halides [115].

In 2022, Fernández‐Salas and Alemán reported a Minisci‐type alkylation of *N*‐heteroarenes with alkyl iodides as radical precursors (Scheme 28) [115]. The electrolysis proceeds in an undivided cell, under an air atmosphere in a tetrahydrofuran (THF)‐water solvent mixture in the presence of phosphoric acid promoter (1 eq.). The proposed role of nickel foam in this system is the reduction of molecular oxygen, which leads to the formation of hydroxyl radicals that abstract an iodine atom from alkyl iodide, resulting in the formation of alkyl radicals. The formed radicals add to the protonated electron‐deficient quinoline, and upon oxidation at the RVC anode, the rearomatized functionalized quinoline products are obtained. The method is distinguished by good functional group tolerance, especially toward acid‐sensitive moieties such as Boc‐protected amines or strained THF moieties, and is applicable to late‐stage functionalization of quinolines.

**SCHEME 29:**
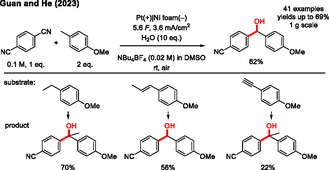
Electrochemical hydroxyarylation of benzylic positions [116].

In 2023, Guan and He reported a system for hydroxylarylation of benzylic positions based on radical–radical cross‐coupling. (Scheme 29) [116]. In this paired electrolysis system, the Ni foam cathode facilitates the reduction of dicyanobenzene to its persistent radical anion. The highlight of the work is that there are different possible precursors of transient ketyl radicals formed on the Pt sheet anode. The authors demonstrated the suitability of all differently hybridized C(sp^3^/sp^2^/sp) benzylic carbon radical precursors: alkyl benzenes, styrenes, and phenylacetylenes, giving desired products in mostly good yields. The mechanism of the transformation varies depending on the type of radical precursor.

In addition to systems in which Ni foam directly participates in reactions with organic molecules, we briefly mention systems where Ni foam functions solely as an efficient catalyst for the hydrogen evolution counter‐reaction, while the organic transformations occur at the anode [117, 118, 119, 120, 121].

## 5 Conclusions

Although nickel foam electrodes have been around for quite some time, they are becoming even more widespread due to their versatile applications. In electro‐organic transformations, these electrode systems provide large surfaces, which is one of the reasons why they advance into technical employment. Nickel foam electrodes offer a unique set of catalytic features that are not met by other high‐surface electrode materials. As an anode material, nickel foam already demonstrated a track record as a versatile electrode material for a broad variety of anodic oxidation reactions, and new applications were found in recent years. Examples are the conversion of biomass, such as lignosulfonates, to value‐added products such as vanillin, which has found application on an industrial scale, or the ring‐opening of cyclohexanols via enolizable ketones as key intermediates. During electrolysis in alkaline aqueous media, an electrocatalytically active surface layer of nickel oxide hydroxide (NiOOH) is formed and regenerated continuously. For such applications, the pretreatment of the nickel anode is of central importance. As a cathode, nickel foam has emerged as a remarkably versatile material for electro‐organic synthesis. It can be used across a wide range of transformations, including hydrogenations, carboxylations with CO_2_, and metal‐catalyzed and radical cross‐coupling reactions. The demonstrated compatibility with acidic and aprotic media, tolerance of sensitive functional groups, and successful translation from batch‐type setup to multi‐gram scale in flow setup highlight its strong potential for industrial application. At the same time, challenges remain to be addressed, such as control of competing hydrogen evolution or the need for more sustainable counterreactions in some systems. We believe that continued advances in reactor designs, paired electrolysis strategies, and mechanistic understanding will further expand the synthetic utility of nickel foam, positioning it as a key enabling material in the future of sustainable electrochemical synthesis.

We expect that by using this electrode material, actual limitations in electro‐organic synthesis will be overcome and novel electrochemical transformations will be realized. In particular, exploration of these electrodes in combination with solvation science will provide new avenues. We also anticipate a growing number of publications and patents on the use of nickel foam in technologically relevant electrosynthetic transformations, with impacts expected to extend beyond academic laboratories into practical and industrial applications.

## Author Contributions

S.R.W. conceived the idea for the review. R.N. and J.S. conducted the literature search and drafted the initial manuscript. S.R.W. supervised the project and edited the manuscript. All authors reviewed and approved the final manuscript.

## Funding

This study was supported by Bundesministerium für Bildung und Forschung (FKZ 03ZU1205AC, FKZ 03ZU1205IA and FKZ 03ZU1205IB), Deutsche Forschungsgemeinschaft (EXC‐2033–390677874—RESOLV).

## Conflicts of Interest

The authors declare no conflicts of interest.
